# Agitation – a case study highlighting the importance of the IPA criteria

**DOI:** 10.1002/alz70857_104455

**Published:** 2025-12-25

**Authors:** Zahinoor Ismail

**Affiliations:** ^1^ Hotchkiss Brain Institute, University of Calgary, Calgary, AB, Canada

## Abstract

Agitation in Alzheimer disease and related dementias (ADRD) is associated with poorer function, loss of independence, risk of hospitalization and/or transfer to higher levels of care, use of pharmacotherapy, accelerated progression to severe dementia and death, higher health care costs, poorer quality of life, and substantial caregiver burden. Despite presenting in up to 60% of persons with AD, agitation‐related behavioural changes are not detected reliably, and often not early enough. This poor and inconsistent detection of agitation has been based, historically, on a symptomatic view of agitation. The literature describes myriad symptoms as agitation, without consistently used standards, providing challenges to meaningful measurement, and consequently meaningful assessment of treatment response. To address this foundational issue in clinical care and research, the International Psychogeriatric Association (IPA) developed and recently validated criteria for Agitation in Cognitive Disorders. The IPA definition describes distressing behaviours in the domains of verbal aggression, physical aggression, and excessive motor activity. This syndromic approach to agitation is an improvement over previous symptomatic descriptors of agitation and helps standardize communication about these dementia‐related behaviours. We present a clinical case incorporating the IPA agitation criteria to demonstrate the clinical benefit of using IPA nosology and nomenclature in assessing and measuring agitation, discussing these behaviours with family and caregivers, and making clinical decisions.

